# Rosmarinic Acid and Flavonoids of the Seagrass *Zostera noltei*: New Aspects on Their Quantification and Their Correlation with Sunlight Exposure

**DOI:** 10.3390/plants12244078

**Published:** 2023-12-06

**Authors:** Isabel Casal-Porras, Kimberly Muñoz, María J. Ortega, Fernando G. Brun, Eva Zubía

**Affiliations:** 1 Departamento de Biología, Facultad de Ciencias del Mar y Ambientales, Universidad de Cádiz, Puerto Real, 11510 Cádiz, Spain; isabel.casal@uca.es (I.C.-P.); kimberly.munoz@uca.es (K.M.); fernando.brun@uca.es (F.G.B.); 2 Departamento de Química Orgánica, Facultad de Ciencias del Mar y Ambientales, Universidad de Cádiz, Puerto Real, 11510 Cádiz, Spain; mariajesus.ortega@uca.es

**Keywords:** phenolic metabolites, seagrasses, drying, freezing, rosmarinic acid, sulfated flavonoids, photoprotection, UV light

## Abstract

Seagrasses are plants adapted to the marine environment that inhabit shallow coastal waters, where they may be exposed to direct sunlight during low tides. These plants have photoprotection mechanisms, which could include the use of phenolic secondary metabolites. In this study, rosmarinic acid (RA) and the flavonoids of *Zostera noltei* from the Bay of Cadiz (Spain) have been analyzed, first to define suitable conditions of leaves (i.e., fresh, dried, or frozen) for quantitative analysis, and then to explore the potential correlation between the phenolic profile of the leaves and sunlight exposure using an in situ experimental approach. Compared with fresh leaves, the contents of RA and flavonoids were significantly lower in air-dried and freeze-dried leaves. Freezing caused highly variable effects on RA and did not affect to flavonoid levels. On the other hand, the content of RA was significantly higher in plants that emerged during low tides than in plants permanently submerged, while plants underneath an artificial UV filter experienced a progressive reduction in RA content. However, the major flavonoids did not show a clear response to sunlight exposure and were unresponsive to diminished UV incidence. The results showed a positive correlation of RA with direct sunlight and UV exposure of leaves, suggesting that this compound contributes to the photoprotection of *Z. noltei*.

## 1. Introduction

Marine angiosperms, commonly known as seagrasses, are a group of about seventy species of plants adapted to the marine environment that can be found forming meadows along worldwide temperate and tropical coasts [1,2]. The ecological importance of seagrasses is well known since they are highly productive ecosystems that contribute to the stabilization of sediments and provide habitat and food to fishes, crustaceans, and mollusks, among others [2]. Moreover, the consensus about the importance of seagrasses in the sequestration of CO_2_, has arisen in recent years a renewed interest for the research on the functioning, conservation, and restoration of these ecosystems as key resources for the development of the Blue Carbon strategy [3,4].

Seagrasses inhabit shallow subtidal and intertidal waters, where they experience high fluctuations of irradiance and may even remain directly exposed to sunlight during low tides [5]. To avoid photodamage, these plants developed mechanisms of protection similar to those of terrestrial plants, including the transient reduction of photosynthetic efficiency [5,6,7] or the xanthophyll cycle [8,9,10]. On the other hand, seagrasses produce secondary metabolites with functions in the defense of the plant and the adaptation to environmental stressors [11,12]. In this regard, a question that has been scarcely explored is the potential involvement of phenolic metabolites, characterized by possessing UV-absorbing and radical-scavenging properties, in the photoprotection of seagrasses, as has been shown for some terrestrial plants [13,14,15].

Among marine angiosperms, the species *Zostera noltei* Hornemann (dwarf eelgrass) is widely distributed along the northeastern Atlantic coasts, from Norway to Mauritania, the Mediterranean, and the Black Sea [1]. From a chemical point of view, although the presence of phenolic acids and flavonoids in *Z. noltei* was detected more than forty years ago [16,17], only in recent years several studies have reexamined the phenolic profile of this species collected in different geographical regions [18,19,20,21,22]. Rosmarinic acid (RA) has been identified in all the collections of *Z. noltei*, together with various of the flavonoids apigenin 7-sulfate (APS), luteolin 7-sulfate (LS), diosmetin 7-sulfate (DS), luteolin 7-glucoside (LG), and apigenin 7-glucoside (APG) [18,19,20,21,22,23]. In some instances, zosteric and caffeic acids have also been reported [18,19,22] and/or minor amounts of the flavonoids acacetin 7-sulfate [20,23], luteolin 7-(6″-malonyl)glucoside [21], and apigenin 7-(6″-malonyl)glucoside [21]. Moreover, three different flavonoid patterns have been described for *Z. noltei*, depending on the geographical region [22].

On the southwestern coasts of Europe, *Z. noltei* thrives in the Bay of Cadiz (Spain) [1], where meadows can be found covering shallow subtidal to upper intertidal areas. These meadows provide the opportunity to obtain clues on the potential role of phenolic compounds in the photoprotection of the plants by comparing the profiles of plants of the same population subjected to different immersion regimes. The characteristic phenolic profile of *Z. noltei* from the Bay of Cadiz consists of RA and APS as major metabolites, although the reported quantitative data differ significantly among studies. Several factors may contribute to these differences, but it is worth noting that the lower reported phenolic contents derive from studies of dried leaves [18,19,22] and higher contents from analyses of fresh leaves [20,23]. In fact, the influence of different drying methods or freezing on the phenolic constituents of plants has already been noted [24]. Moreover, the effects of these pretreatments are not necessarily the same for the different compounds, thus affecting the phenolic profile attributed to the plant and also the ecological conclusions derived from it.

In this context, the first part of this study aimed to determine whether there are differences in the phenolic profile deduced by analyzing fresh, dried, and frozen leaves of *Z. noltei* and thus determine the best procedure for the subsequent studies. The next goal was to investigate whether there is any correlation between the level of exposure to sunlight of *Z. noltei* leaves and their content of phenolic secondary metabolites, which could support a photoprotective role of these compounds. For this purpose, we first performed an in situ study to examine the quantitative phenolic profile of plants that emerged during low tides, which experience direct sunlight exposure, in comparison with surrounding plants thriving in permanently submerged meadows. In addition, an in situ manipulative experiment was designed, which consisted of using UV filters above *Z. noltei* meadows and monitoring the phenolic metabolites for sixty days in UV-shaded and control (non-UV-shaded) plants.

## 2. Results

### 2.1. Analysis of Phenolic Secondary Metabolites in Fresh, Dried, and Frozen Leaves of Z. noltei

**Figure 1:**
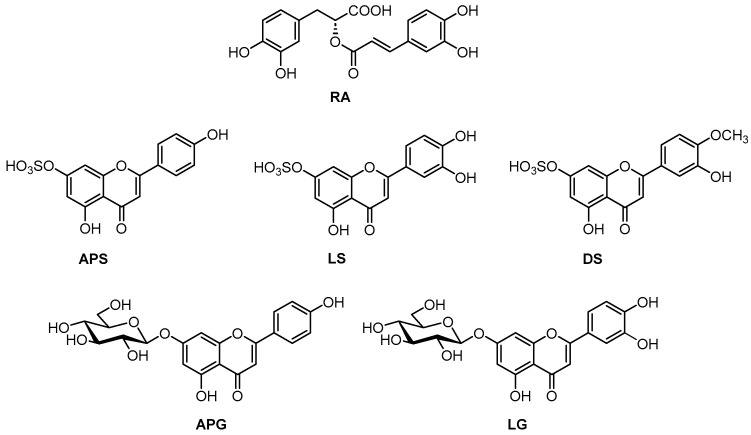
Chemical structures of the phenolic metabolites of *Z. noltei*: rosmarinic acid (RA), apigenin 7-sulfate (APS), luteolin 7-sulfate (LS), diosmetin 7-sulfate (DS), apigenin 7-glucoside (APG), and luteolin 7-glucoside (LG).

The UPLC–MS analysis of phenolic acids and flavonoids in the extracts of fresh leaves of *Z. noltei* from different sites of the Bay of Cadiz (Figure S1) showed that rosmarinic acid (RA) (Figure 1) was always the major phenolic compound of the leaves, with contents in the range 22–42 mg/g dw of leaves (Table 1). The flavonoid content was dominated by APS, with levels in the range of 11–17 mg/g dw, followed by another two sulfated derivatives, LS and DS, and the glycosilated compounds APG and LG. The total flavonoid contents found were in the range of 20–30 mg/g dw. Trace amounts of acacetin sulfate were also detected.

**Figure 2:**
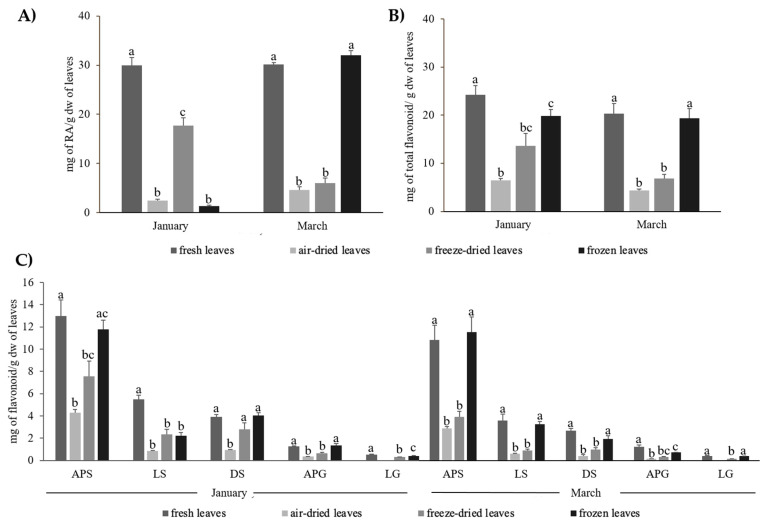
Content of phenolic metabolites in the leaves of *Z. noltei* (mg/g dw) determined by extraction of fresh, air-dried, freeze-dried, or frozen leaves; (**A**) RA content; (**B**) total flavonoid content; (**C**) individual flavonoid (APS, LS, DS, APG, LG) content. Data are presented as the mean ± SE (*n* = 3). For each compound, different letters indicate significant differences (*p* < 0.05, Table S1).

The contents of RA and total flavonoids in the leaves of *Z. noltei* determined from the extracts of fresh leaves, together with those derived from leaves of the same collection that had been air-dried, freeze-dried, or frozen prior to extraction, are shown in Figure 2A,B and Table S2. Air-drying of leaves caused significant decreases in the concentration measured both for RA and the flavonoids, although the effects were more marked on RA. For the first collection (January), air-dried leaves yielded a content of RA 91.7% lower than the RA concentration determined by analysis of the corresponding fresh leaves. Despite freeze-drying caused a lower effect, the yield of RA obtained was still 41.1% lower than the level found in fresh leaves. The analysis of frozen leaves yielded a strong drop in the RA content (95.5%) with respect to fresh leaves. These results were further supported by analyzing a second collection (March), which also yielded significantly lower RA contents in air-dried and freeze-dried leaves than in fresh leaves (84.9% and 80.1% decreases, respectively). However, an important discrepancy was observed for frozen leaves, whose content in this second collection was similar to that of fresh leaves. A discussion on this issue can be found in Section 3.1.

The total flavonoid content measured in air-dried and freeze-dried leaves of *Z. noltei* in the first collection examined was 73.4% and 43.6% lower than the level obtained from fresh leaves, respectively. In the analysis of the second collection (March), decreases in the total phenolic content upon air-drying and freeze-drying were again observed, even more marked (78.5% and 66.6%, respectively). On the other hand, in both collections, the total flavonoid content obtained from frozen leaves did not significantly differ from that derived through analysis of fresh leaves. The contents of the individual flavonoids determined from fresh or pretreated leaves are shown in Figure 2C. All flavones followed a similar trend, with the lowest levels measured in air-dried leaves (decreases in the range of 67.0–88.1%) and less marked or similar decreases in freeze-dried leaves, while frozen leaves yielded similar contents to fresh leaves.

### 2.2. Phenolic Secondary Metabolites in Z. noltei Leaves Periodically Emerged during Low Tides vs. Leaves Permanently Submerged

The inner part of the Bay of Cadiz includes an area where *Z. noltei* extends continuously from the subtidal to the upper intertidal zone. The contents of the different phenolic metabolites measured in plants of the intertidal zone, which emerge at low tides (Zone I), and in plants of the subtidal, always submerged (Zone S), are shown in Table 2. The quantitative phenolic profile followed the same trend in plants from both zones, with RA as the major metabolite of the leaves, followed by APS, LS, DS, APG, and LG. The analysis of the collection in March showed that leaves from Zone I, which were exposed to direct sunlight during low tides, had as much as 69.5% higher RA content than leaves from Zone S. The total flavonoid content was also 47.9% higher in leaves from Zone I. In fact, every flavonoid was significantly more abundant in leaves from Zone I, except LS. The level of the major flavonoid APS was 35.2% higher in leaves from Zone I. The minor flavonoid APG exhibited the highest difference, and its content was 2.4-fold higher in the leaves of Zone I. The results obtained from a second collection performed in April showed the same trend, although the differences between zones were less marked, with 25.4% and 15.3% higher levels of RA and flavonoids, respectively, in leaves from Zone I. LG exhibited the highest differences among zones, but the levels of APS remained unchanged.

### 2.3. Phenolic Secondary Metabolites in Z. noltei Plants Shaded with UV Filters

**Figure 3:**
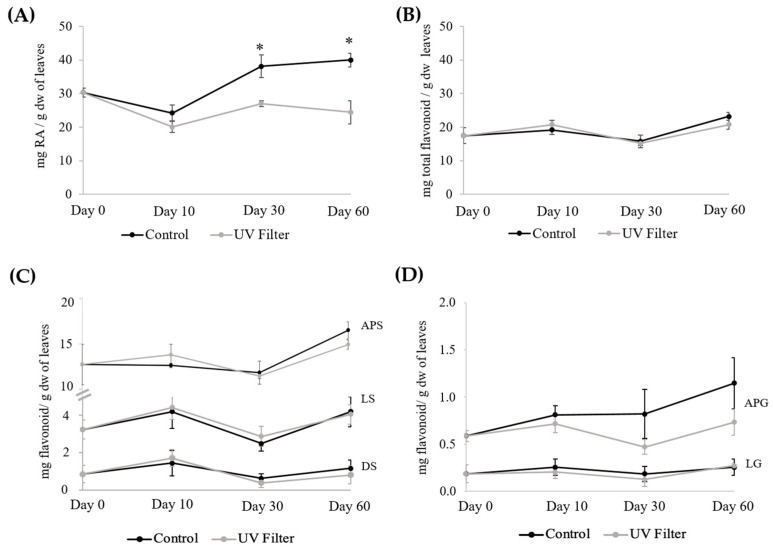
Contents (mg/g dw) of (**A**) RA, (**B**) total flavonoid, (**C**) individual sulfated flavonoids, and (**D**) individual flavonoid glycosides in the leaves of *Z. noltei* under a UV filter for sixty days; please note the different scales used in graphics (**C**,**D**). Data are presented as the mean ± SE (*n* = 4). For each day and compound, an asterisk means a significant difference (*p* < 0.05, Table S1).

In order to gain evidence about the potential effect of sunlight, in particular the UV component, on the content of phenolic metabolites in *Z. noltei*, an in situ manipulative experiment was performed. In a meadow located in a shallow area with a smooth bottom and no significant differences in depth within the seagrass meadow, four areas in the meadow were covered with a plastic UV filter, and the phenolic compounds of the plants under the filter were analyzed on days 10, 30, and 60 (Figure S2). The results obtained, together with those of the corresponding controls (adjacent plants not covered by the filter), are shown in Figure 3 and Table S3. After 10 days, it was already noted that there was a slight decrease in the content of RA in plants under the filter. After 30 days, the content of RA had decreased by 29.1%, and after 60 days, the content of RA in plants under the filter was only 61.0% of that found in control plants. On the other hand, the flavonoid content of leaves under the filter did not differ from that found in control leaves.

### 2.4. UV Spectra and Antioxidant Activity of Extracts

**Figure 4:**
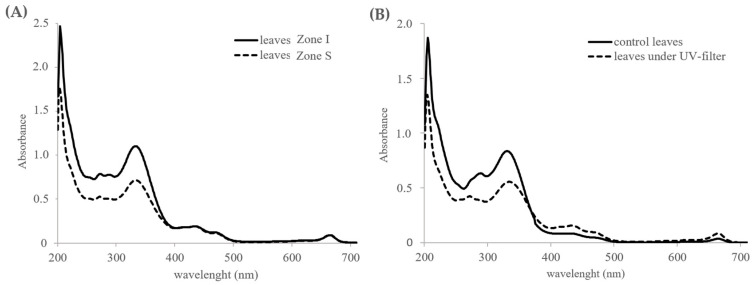
UV–Vis spectra of methanolic extracts of (**A**) leaves of plants emerged during low tides (Zone I) and from plants permanently submerged (Zone S) and (**B**) leaves after sixty days under a UV filter and control leaves.

The photoprotective capacity of phenolic acids and flavonoids lies in the capability of these compounds to act as screens that absorb UV radiation and their antioxidant properties through scavenging reactive oxygen species (ROS) formed during photochemical stress. The UV spectra of the methanolic extracts derived from fresh leaves of *Z. noltei* exhibited a clear maximum absorption at 330 nm, attributable to their high content in RA (maxima at 292 and 331 nm) (Figure S3). As shown in Figure 4, the extracts of plants from the intertidal zone (Zone I), with high RA content, showed higher UV absorbance than those of subtidal plants (Zone S). On the other hand, the extracts of plants located for sixty days under a UV filter, whose RA content had decreased, showed a lower capacity for absorbing UV radiation than the extracts of control plants.

Regarding the antioxidant capacity, a positive correlation was found between the content of RA and the radical-scavenging capacity of the extracts (Table 3). The extracts of leaves from plants in the intertidal zone also exhibited higher radical-scavenging activity than the extracts of plants always submerged, with EC_50_ values of 11.69 μg/mL and 27.54 μg/mL, respectively, in the ABTS assay. The antioxidant capacity of extracts of leaves after sixty days under a UV filter was almost half of that exhibited by control plants (EC_50_ 31.19 μg/mL vs. 17.84 μg/mL).

## 3. Discussion

### 3.1. Influence of Drying and Freezing on the RA and Flavonoid Content of Z. noltei Leaves

This study on the phenolic metabolites of *Z. noltei* has evaluated the influence of the pretreatment of leaves (i.e., air-drying, freeze-drying, and freezing) on the resulting phenolic quantitative profile and explored plants under different levels of sunlight exposure together with those artificially protected from UV radiation, providing new data for chemoecological considerations about phenolic metabolites and photoprotection of seagrasses.

The quantitative phenolic profiles obtained for fresh *Z. noltei* leaves collected at different locations of the Bay of Cadiz are consistent with the presence in this region of a single phenolic chemotype, characterized by containing the phenolic acid RA and the sulfated flavonoid APS as major metabolites (22–42 mg/g dw and 11–17 mg/g dw, respectively), together with a series of less abundant flavonoids LS, DS, APG, and LG (total flavonoid 20–30 mg/g dw). A recent study has already indicated that a distinctive feature of *Z. noltei* of the Bay of Cadiz is the presence of APS as major flavonoid [22]. However, the contents of phenolic compounds described in that study (10.52 mg/g dw of RA, 6.87 mg/g dw of APS, and 10.15 mg/g dw of total flavonoids) and in other reports [18,19] were significantly lower than those measured in our samples. In this regard, the current study has shown that performing the analysis either with fresh or dried leaves of *Z. noltei* strongly influences the quantitative outcome, with different effects on RA and flavonoid contents. It is well established that after the collection of plant material, phenolic compounds may experience irreversible changes, such as oxidative transformations, both non-enzymatic and mediated by polyphenol oxidase [24]. This enzyme, primarily responding to wounding, in the presence of oxygen catalyzes the hydroxylation of phenols to catechols and the oxidation of these to *o*-quinones, which non-enzymatically decompose or polymerize into melanin pigments associated with the browning of wounded tissues [25,26]. Thus, it has been proposed that for quantitative analysis of phenolic compounds, samples should be extracted fresh immediately after collection [24,27]. Accordingly, studies of a variety of plants and fruits have described significant losses of phenolic compounds upon drying of samples, although the effects differ among compounds [27,28,29,30,31,32]. Nonetheless, other authors have advised the use of dry, frozen, or freeze-dried samples to avoid degradation by enzyme action [33,34]. In this study, we have shown that the content measured for every phenolic metabolite in the air-dried leaves of *Z. noltei* was significantly lower than the value obtained from fresh leaves of the same collection. RA was the compound most sensitive to air-drying, showing decreases up to 91.7%, while losses up to 78.5% were observed for the total flavonoid content. Even when applying heat for extraction, the RA and flavonoid contents were still significantly lower than those of fresh leaves. It is also worth noting that these degradative processes, whose extension is difficult to control once started, explain the variability of quantitative data between the collections analyzed. Moreover, the results of this study suggest that the phenolic contents previously reported for *Z. noltei* could have been underestimated since they were obtained by analyzing air- or oven-dried leaves [18,19,22]. Our results are in line with the decrease of about to the half of RA and total flavonoid contents reported for rosemary leaves after air-drying [29]. Although the loss of water upon drying of plant samples causes inhibition of enzymes such as polyphenol oxidase, the decrease of enzymatic activity is slow during air-drying, and degradation of phenolic metabolites may occur before the plant material is completely dried [35]. Using oven- or hot air-drying accelerates enzymatic inactivation, leading, in some instances, to higher contents than air-dried samples [35] and, in others, to lower contents attributable to thermal degradation [28]. The freeze-drying of *Z. noltei* leaves caused similar or less marked decreases in RA and flavonoid contents than air-drying, as has also been described in studies of other plants [29,30,31,36,37].

On the other hand, freezing is a method often used for the conservation and storage of plant samples prior to analysis. In this study, the analysis of the first collection of frozen leaves of *Z. noltei* showed a drastic loss of RA and no significant decrease in flavonoids. Similar results have been previously described in rosemary leaves (65–80% loss of RA, 11.5% of flavonoids) [29]. The high decrease of phenolics upon freezing may be due to the tissue damage caused by ice crystals, which leads to the loss of cellular integrity and the exposure of compounds to oxidative enzymes, resulting in quick degradation during storage or thawing [38,39]. This proposal was supported by the results obtained in the analysis of the second collection, where frozen leaves were directly treated with the extraction solvent to avoid thawing, resulting in an RA content similar to that of fresh leaves.

Taken together, the results of this study strongly support that for quantitative analysis of phenolics in *Z. noltei*, the samples should be analyzed immediately since drying, and sometimes freezing, of the samples could lead to underestimating the content of these metabolites in the plant and also to obtaining inaccurate ratios among the different compounds.

Among marine angiosperms, a study of the influence of tissue handling on the flavonoids of *Posidonia oceanica* also showed that flavonoids were significantly reduced after freeze-drying and chilling. However, similar contents were reported for fresh and oven-dried leaves, which was attributed to quick drying of entire leaves, which minimized the enzymatic decomposition [40].

### 3.2. RA and Flavonoid Content in the Leaves of Z. noltei Plants Subjected to Different Sunlight Exposure

Seagrasses in the intertidal and subtidal zones are under highly different light conditions. In particular, plants in the upper intertidal areas are regularly emerged and exposed to oversaturating irradiances because of the direct incidence of sunlight, while irradiance reaching plants in the subtidal area is attenuated by the water column and suspended particles [5,7]. It is worth noting that the measurement of the concentrations of the different secondary metabolites of a plant, as well as their variations under different biotic and abiotic conditions, provide valuable clues on the function that those metabolites may exert. In this regard, despite the distribution of *Z. noltei* in intertidal and subtidal zones, the differences among the phenolic profiles of plants from both zones of a meadow have never been studied. We have found that within the same meadow, the content of RA in plants of the intertidal zone was up 69.5% higher than the content in plants of the subtidal zone, suggesting a role of RA in the photoprotection of the plant. These results are consistent with data reported in studies of terrestrial plants where the accumulation of RA and other phenolic compounds was observed upon exposure of leaves and other parts of the plant to different light treatments [41,42,43,44,45]. Although other stress factors, such as dryness, could also contribute to the higher level of RA in periodically emerged plants, further support for the proposed role of RA in *Z. noltei* was obtained through the in situ experiment on plants with the same emersion regimen. This experiment revealed a progressive decrease of RA in plants artificially protected from solar UV radiation with a filter, with the RA content reduced to 61.0% after sixty days. Moreover, this result suggests that the synthesis or accumulation of RA in *Z. noltei* specifically correlates with the UV component of sunlight radiation.

The photoprotective activity of phenolic metabolites lies in their capability to act as screens that absorb UV radiation and their antioxidant properties through scavenging ROS formed during photochemical stress [13,46]. In agreement with the levels of RA measured, the UV absorbance and radical-scavenging activity of the extracts of leaves from the intertidal zone were higher than those of extracts from the subtidal zone, indicating that these plants have adapted to cope with the effects of direct sunlight exposure. Moreover, the low UV absorbance and radical-scavenging activity of extracts of leaves covered by a UV filter for sixty days correlated with the decrease in RA level.

Taken together, the results show for the first time in seagrasses a positive correlation between exposure to sunlight and a phenolic acid, RA, whose level seems to be modulated to cope with sunlight variations, in particular with UV radiation, suggesting that RA has a photoprotective role in the plant.

On the other hand, the total flavonoid content in *Z. noltei* plants of the intertidal zone was significantly higher than that found in plants of the subtidal zone. Flavonoids are known to play a variety of functions in the relationship of plants with the environment, including photoprotection [46,47]. In this regard, diverse evidences support that the photoprotection provided by flavonoids mainly lies in their activity as scavengers of the ROS generated during light stress rather than acting as screens [14,15,46,48]. Thus, it has been reported the accumulation of *o*-dihydroxylated B-ring flavonoids (e.g., luteolin and its 7-*O*-glucoside), but not of those with a single hydroxy group on ring B (e.g., apigenin and its 7-*O*-glycosides, luteolin 4′-*O*-glucoside) in plants exposed to high levels of sunlight or UV irradiance [46,49,50,51]. Nonetheless, it was also pointed out that an additional *o*-hydroxy group in the B-ring does not affect the UV-absorbing properties but enhances the antioxidant capacity [15,48]. These data, together with the location of flavonoids close to centers of ROS generation in stressed cells, have led to the proposal that the main role of flavonoids in plant photoprotection is reducing photo-oxidative damage [15,48]. In this study, higher total flavonoid content was found on leaves with direct sunlight exposure, but, differing from previous reports, we observed significantly higher levels of flavonoids both with *o*-dihydroxylated B-rings (LS, LG) and monohydroxylated B-rings (APS, DS, and APG). Although the higher differences between leaves exposed to sunlight and submerged were found for APG and LG, these are minor metabolites whose potential contribution to UV absorption and radical scavenging is expected to be very limited in comparison with that of the much more abundant analogs APS and LS. In this regard, the response observed for these compounds was unclear since sunlight-exposed leaves contained a higher content of APS only in one collection and a moderately higher content of LS in the other. Moreover, taking into account the higher antioxidant, and hence protective, capability of lutein derivatives with respect to apigenin derivatives [48], the increased levels of APS and LS seem to be due to other factors beyond high light incidence. On the other hand, during the sixty days of the in situ experiment, the level of every flavonoid remained unchanged in plants receiving sunlight devoid of UV radiation. These data are consistent with studies showing that UV radiation is not a prerequisite for flavonoid biosynthesis [49]. Other studies reporting unchanged levels of dihydroxylated B-ring flavonoids (luteolin and orientin) in various grasses exposed to different UV-B intensities have proposed that constitutive levels of the flavonoids were high enough to protect the leaves against ambient and higher levels of UV-B radiation [52].

Taken together, the higher levels of flavonoids in *Z. noltei* leaves under sunlight exposure and the lack of relationship with UV radiation observed in this study suggest that although these compounds could eventually contribute to attenuate photodamage, their main role might be in response to other factors. It is worth noting that hydroxycinnamic acid derivatives (e.g., RA) exhibit UV absorption maxima in the 290–320 nm region and are better attenuators of UV-B radiation than flavonoids, whose absorption maxima are often beyond 340 nm [15,48]. In this study, we found that plants exposed to direct sunlight contained higher concentrations of RA, whose maximal absorptions at 292 and 331 nm may be an efficient screen for the deleterious UV-B component of solar radiation. In addition, RA may also provide antioxidant protection because of its high radical-scavenging properties. Therefore, although RA and some of the flavonoids (e.g., LS) might exert similar functions in photoprotection, in this study, only the phenolic acid RA was clearly responsive to changes in sunlight and UV exposure of *Z. noltei* leaves. Different results have been reported for some species where, upon high light, the content of hydroxycinnamic acid derivatives remains unchanged and the ratio of hydroxycinnamates to flavonoids decreases, suggesting the predominant role of flavonoids in the photoprotection of those plants [46,50,53]. Therefore, it seems that there is not a common trend in plants containing both, phenolic acids and flavonoids, regarding to the utilization of these classes of metabolites in the responses to light stress. Some species have flavonoids as preferential photoprotective agents [46,50,53], and in others, like *Z. noltei*, this role seems to be attributed to the phenolic acids.

In the marine environment, RA and sulfated flavonoids have also been described from *Z. marina* [21,54,55,56]. A recent study of *Z. marina* plants from Norwegian coasts has found a moderate positive correlation of RA concentration with depth and a strong negative correlation for flavonoids, which has been interpreted as a lack of stress conditions with higher depth rather than a different response to light conditions [54]. In our study, the responses of flavonoids suggest that they could play other roles beyond photoprotection. In fact, we have recently indicated their contribution to the chemical defense of *Z. noltei* against herbivores based on the deterrent properties of the natural mixture of RA and flavonoids against the sea urchin *Paracentrotus lividus* [23]. An antifouling function could also be expected for the flavonoids of *Z. noltei*, taking into account the activity recorded for RA, LS, and DS as inhibitors of the settlement of bacterial microfoulers [56]. It is also worth noting that most of the flavonoids of *Z. noltei*, including those most abundant, exhibit the hydroxy group at C-7 esterified as sulfate, which improves the water solubility. The formation of sulfate esters has been considered an adaptation of plants growing in aquatic environments rich in mineral salts [57]. Although the purpose of the sulfation of flavonoids in seagrasses is not clear, it has been suggested that it could be a mechanism of detoxification from sulfide intrusions [22] or play a physiological function such as facilitating the transport or storage of bioactive compounds [55].

## 4. Materials and Methods

### 4.1. Sampling Sites and Plant Materials

#### 4.1.1. *Zostera noltei* Hornemann

*Zostera noltei* (Hornemann, 1832) is considered a pioneer and fast-growing species, displaying clonal growth with plagiotropic (i.e., horizontal) rhizomes bearing shoots and roots at every node, while vertical shoots (i.e., orthotropic) can also be found under burial stress [58]. This species inhabits shallow subtidal and intertidal areas till 2–3 m depth, developing crowed populations up to 20,000 shoots m^−2^ [59].

#### 4.1.2. Samples for the Study of Phenolic Profile in Fresh, Dried, and Frozen Leaves

Leaf samples of *Z. noltei* for this study were collected in the Bay of Cadiz, an Atlantic shallow environment located in the SW of Spain (Figure S1). This bay is formed by two water bodies (external and internal) well communicated by tides through a narrow strait. Meadows of *Z. noltei* can be found along the inner bay from the subtidal to the upper intertidal zone. Sampling was performed in two sites of the inner part of the Bay: site 1 at 36°28′5′ N, 6°15′11″ W (January, March) and site 2 at 36°29′22′ N, 6°15′48″ W (March). Samples were also collected in a tidal earthen pond (36°30′48″ N, 6°9′53″ W) connected to the inner bay (May, June) (site 3). Intact vertical shoots [60] were carefully collected and transported to the laboratory in a thermal refrigerator. The collected shoots were washed with fresh water to remove epiphytes and organic and inorganic debris, and they were immediately extracted. For the collections of January and March, a portion of the leaves (three replicates of 3 g) were extracted immediately, while the remaining were separated into three groups for different treatments before extraction (three replicates of 3 g in each group): air-drying in the shade at room temperature (r.t.) until constant weight, freeze-drying, and freezing at −20 °C. The water content of leaves was 78.0 ± 0.8% (*n* = 3) on a fresh weight basis.

#### 4.1.3. Samples for the Study of Phenolic Profile in Leaves Periodically Emerged vs. Leaves Permanently Submerged

Samples were collected in March and April at site 2, where plants are continuously extended in a shallow area at 0.4 and −0.8 m above and below the chart datum (lowest astronomical tide). Leaves of plants of the upper intertidal zone, which emerge during low tides, were collected at 36°29′20.6″ N, 6°15′58.3″ W. The average emersion period of plants was 6 h per day during the months of March and April. Samples of plants from the same meadow but always submerged were collected at 36°29′21.7″ N, 6°15′48.1″ at a depth of 0.6 m during low tide. The collected shoots (0.5 g) were washed with fresh water to remove epiphytes and organic and inorganic debris and immediately extracted.

### 4.2. Experimental Design and Sampling for the Experiment In Situ on the Influence of Exposure to Sunlight

The experiment was performed in a tidal earthen pond of 52 × 16 m (36°30′48″ N, 6°19′53″ W), which harbors a healthy and abundant population of *Z. noltei*. Square areas (60 × 60 cm) of the meadows were shaded with a colorless PVC film with UV-excluding properties (D-c-fix, 99% UV-blocking capacity, light transmittance 80%). The film was attached to a solid frame of 60 × 60 × 30 cm, which was anchored to the bottom, allowing the free flow of water under the frame (Figure S2). Control treatments consisted of allocating the same solid frame but without the UV filter. In all cases, four experimental plots (i.e., control and UV filter) were allocated randomly in the meadow. The experiment lasted sixty days. Each ten days the film was cleaned to remove deposited sludge. Initial samples of leaves were collected before placing the filters. After the beginning of the experiment, samples of leaves (10–20 shoots) were collected both from plants under the filter (leaves collected in the central part of the experimental square) and control plants on days 10, 30, and 60 and immediately extracted.

### 4.3. Extraction

For samples collected and pretreated as indicated in Section 4.1.2 (3 g of fresh leaves and materials derived from 3 g of fresh leaves by air-drying, freeze-drying, or freezing), 30 mL of methanol were added to each sample at r.t. Then, the plant material was mashed with a pestle, and the mixture was placed in an ultrasound bath for 5 min. The solution was filtered by gravity over paper at r.t., and the residual plant material was extracted two more times following the same procedure. The solutions obtained were combined, and the solvent evaporated to dryness in a rotary evaporator. The same procedure was used for the extraction of fresh leaves collected as indicated in Section 4.1.3 and Section 4.2, using 3 mL of methanol for each 100 mg of plant material.

### 4.4. Quantitative Analyses by UPLC–MS

A portion of the extract (25–30 mg) was suspended in 2 mL of methanol/H_2_O 8:2 (*v*/*v*) and transferred onto an SPE-C18 cartridge (0.5 g/3 mL) (Supelco, Bellefonte, PA, USA) preconditioned with 1 mL of the same solvent. The cartridge was eluted with 7 mL of methanol/H_2_O 9:1 (*v*/*v*), and the obtained solution was evaporated in a rotary evaporator. The residue was dissolved in methanol and filtered over 0.22 μm. Samples for UPLC–MS were prepared with final concentrations of 50 μg/mL or 125 μg/mL of extract and spiked with quercetin as an internal reference to reach 1.125 μg/mL.

The analyses were performed on an Acquity UPLC H-Class (Waters, Milford, MA, USA) coupled to a XEVO G2 Q-TOF spectrometer equipped with an electrospray ionization source (ESI) (Waters, Manchester, UK) (Figure S4). The chromatography was achieved on an Acquity UPLC HSS T3 column (100 × 2.1 mm, 1.8 μm). The mobile phase was composed of (A) H_2_O + 0.1% formic acid and (B) acetonitrile + 0.1 formic acid, infused at 0.6 mL/min. The elution program was: 0–0.5 min 90% A (isocratic), 0.5–3.2 min 40%A (linear gradient), 3.2–3.5 min 100% B (linear gradient), 3.5–4.0 min 90% A (linear gradient), 4.5–5.0 min 90%A (isocratic). The mass spectrometer operated in the negative ion mode (ESI(−)), full scan acquisition. The parameters were source temperature 120 °C, capillary voltage 3 kV, and mass scan range *m*/*z* 100–1000 Da. For accurate mass measurements, the reference lock spray mass as the negative ion of leucine-enkephalin (*m*/*z* [M-H]^−^ 554.2615) was used. The quantification of the phenolic compounds was performed using external calibration curves. For the quantification of RA, a calibration curve in the range 0.5–15.0 μg/mL (r^2^ = 0.9907) was constructed with an RA standard (Sigma, St. Louis, MO, USA). Since there is no commercially available any sulfated flavonoid to be used as a standard, pure APS previously isolated from *Z. noltei* in our laboratory [20] (Figure S5) was used to construct a calibration curve in the range 0.5–7.5 μg/mL (r^2^ = 0.9972) for the quantification of APS, LS, and DS. For the quantification of LG and APG, the flavonoid glycoside rutin (Sigma, St. Louis, MO, USA) was used as a standard, based on the principle of structure-related target analyte/standard, and a calibration curve in the range 0.1–3.0 μg/mL was constructed (r^2^ = 0.9965). The data obtained from LC–MS were subsequently entered into MassLynx V4.2 software. The method of quantification was validated by considering the linearity, the limits of detection and quantification, and the intraday and interday variability (RSD < 5%).

### 4.5. UV Spectra

The UV–Vis spectra of solutions in ethanol of extracts at 100 μg/mL and of RA (Sigma, St. Louis, MO, USA) were recorded using a VWR 1600 PC spectrophotometer (VWR, Radnor, PA, USA).

### 4.6. Antioxidant Activity

Antioxidant activity was determined by the ABTS (2,2′-azinobis(3-ethylbenzo-thiazoline-6-sulphonic acid) assay developed by Re et al. [61], with slight modifications. In brief, a solution of the radical cation ABTS^·+^ was prepared by mixing (1:1, *v*/*v*) a solution of ABTS diammonium salt (7 mM) and a solution of potassium persulfate (2.45 mM) in H_2_O. The mixture was kept in the dark at room temperature for 12–18 h before use. Then, the solution was diluted with EtOH to an absorbance of 0.70 ± 0.02 at 734 nm. Stock solutions of Trolox standard (Sigma, St. Louis, MO, USA), of RA (Sigma, St. Louis, MO, USA), and of the corresponding extracts were prepared in EtOH. For the assay, 100 μL of the Trolox solution, RA solution, or extract solution were mixed with 2 mL of the ABTS^+^ solution, and the absorbance at 734 nm was measured after 6 min. Controls were prepared by adding 100 μL of EtOH to 2 mL of ABTS^+^ solution. All determinations were carried out in triplicate. The percentage of inhibition of the absorbance was calculated with the following equation: % Inhibition = [(A_0_ − A_1_)/A_0_] × 100, where A_0_ expresses the absorbance of the control and A_1_ is the absorbance of the tested sample.

### 4.7. Statistical Analyses

The effects of drying or freezing leaves, sunlight exposure, and UV radiation on the contents of phenolic compounds in *Z. noltei* leaves were examined using one-way ANOVA. Before the analyses, all data were tested for normality (Shapiro–Wilk normality test) and homoscedasticity (Barlett’s test for homogeneity of variances). If the data were not normal or homoscedastic, a nonparametric Kruskal–Wallis test was applied. Pairwise comparisons to identify homogenous groups were identified using Tukey’s multiple comparisons. Data are presented as mean ± SE. All statistical analysis was conducted on R statistical software 4.0.2 (R Development Core Team 2020) (Table S1).

## 5. Conclusions

This study has shown that for quantitative analysis of the phenolic metabolites of the seagrass *Z. noltei*, the samples should be analyzed immediately after collection since air-drying, freeze-drying, or freezing of the samples may lead to underestimating the content of these metabolites in the plant and obtaining inaccurate ratios among compounds. Moreover, these observations could be applicable to the analysis of phenolic compounds in related species of seagrasses. On the other hand, the quantitative phenolic profile of *Z. noltei* leaves exposed to direct sunlight during low tides displayed, with respect to submerged plants, higher levels of the efficient UV-absorbing and antioxidant compound RA, suggesting a photoprotective role for this phenolic metabolite. Furthermore, the gradual decrease in the levels of RA in plants under UV filters supports the capability of *Z. noltei* to adjust the synthesis or accumulation of this compound upon sunlight variations. However, the major flavonoids did not show a conclusive response to sunlight exposure and were unresponsive to diminished UV incidence, likely reflecting the complexity of the underlying mechanisms that regulate the involvement of these compounds in different defensive functions. Future studies will aim to progress in the knowledge of the diverse mechanisms participated by secondary metabolites in *Z. noltei* and related seagrasses, as well as their importance in a climate change scenario for these plants and the functions and services they provide both to humans and marine ecosystems.

## Figures and Tables

**Table 1:** Content (mg/g dw) * of rosmarinic acid (RA), the sulfated flavonoids APS, LS, and DS, and the flavonoid glycosides LG and APG in fresh leaves of *Z. noltei* collected in three sites of the Bay of Cadiz. Data are presented as the mean ± SE (*n* = 3).

	Site 1	Site 1	Site 2	Site 3	Site 3
Compound	January	March	March	May	June
RA	29.99 ± 1.59	30.12 ± 0.40	24.75 ± 1.72	22.29 ± 1.73	41.58 ± 1.87
APS	12.98 ± 1.46	11.74 ± 1.42	16.49 ± 0.71	12.38 ± 0.29	16.09 ± 1.26
LS	5.51 ± 0.36	3.88 ± 0.62	6.72 ± 0.61	4.40 ± 1.23	3.77 ± 0.96
DS	3.93 ± 0.20	2.92 ± 0.20	4.00 ± 0.43	1.72 ± 0.89	1.12 ± 0.61
APG	1.26 ± 0.04	1.33 ± 0.20	1.78 ± 0.28	0.75 ± 0.11	1.19 ± 0.38
LG	0.53 ± 0.03	0.45 ± 0.03	0.54 ± 0.02	0.23 ± 0.11	0.24 ± 0.12
Total flavonoids	24.22 ± 1.96	20.31 ± 2.11	29.51 ± 1.76	19.48 ± 2.01	22.41 ± 1.20

* dw = dry weight of leaves.

**Table 2:** Content (mg/g dw) of rosmarinic acid (RA) and the flavonoids APS, LS, DS, APG, and LG in fresh leaves of *Z. noltei* collected in March and April from the intertidal (Zone I) and the subtidal zones (Zone S). Data are presented as the mean ± SE (*n* = 3). For each compound within a collection, different letters indicate significant differences (*p* < 0.05, Table S1).

	Compound	Zone I	Zone S
March	RA	41.78 ± 1.59 ^a^	24.65 ± 1.16 ^b^
	APS	22.00 ± 1.16 ^a^	16.27 ± 0.81 ^b^
	LS	7.65 ± 0.27 ^a^	6.96 ± 0.36 ^a^
	DS	7.35 ± 0.21 ^a^	3.96 ± 0.26 ^b^
	APG	4.59 ± 0.40 ^a^	1.94 ± 0.16 ^b^
	LG	1.49 ± 0.06	nq
	Total flavonoids	43.08 ± 1.83 ^a^	29.13 ± 1.38 ^b^
April	RA	47.55 ± 0.66 ^a^	37.92 ± 1.80 ^b^
	APS	21.80 ± 0.41 ^a^	20.66 ± 1.15 ^a^
	LS	7.28 ± 0.42 ^a^	5.84 ± 0.40 ^b^
	DS	4.30 ± 0.09 ^a^	3.67 ± 0.33 ^a^
	APG	4.85 ± 0.26 ^a^	3.63 ± 0.25 ^b^
	LG	1.64 ± 0.11 ^a^	0.80 ± 0.03 ^b^
	Total flavonoids	39.88 ± 0.42 ^a^	34.60 ± 1.73 ^b^

nq: not quantified, below the limit of quantification.

**Table 3:** Antioxidant activities of extracts of leaves of *Z. noltei* and RA in the ABTS assay. Zone I: Extracts of leaves from the intertidal zone; Zone S: Extracts of leaves from the subtidal zone; UV filter: Extracts of leaves after sixty days under a UV filter; Control: Extracts of leaves out of the UV filter, used as control in the experiment. Trolox was used as a positive control for the antioxidant assay. Data are presented as the mean ± SE (asterisk indicates significant differences between Zone I and Zone S, *n* = 3; hash indicates significant differences between UV filter and control, *n* = 4, *p* < 0.05).

	Zone I	Zone S	Control	UV Filter	RA	Trolox
**EC_50_ (μg/mL)**	11.69 ± 0.03 *	27.54 ± 2.66	17.84 ± 3.49 ^#^	31.19 ± 2.83	2.44 ± 0.01	2.45 ± 0.01

## Data Availability

Data are contained within the article and Supplementary Materials.

## Supplementary Materials

The following supporting information can be downloaded at: https://www.mdpi.com/article/10.3390/plants12244078/s1, Figure S1: Map of the Bay of Cadiz (Spain) and sites of *Z. noltei* sampling. Table S1: Statistical data. Table S2: Contents of phenolic metabolites (mg/g dw) in fresh, air-dried, freeze-dried, and frozen leaves of *Z. noltei*. Figure S2: Scheme of the in situ experiment with *Z. noltei*. Table S3: Contents of phenolic metabolites (mg/g dw) in leaves of *Z. noltei* under a UV filter during 10, 30, and 60 days. Figure S3: UV–Vis spectra of a methanolic extract of *Z. noltei* and rosmarinic acid (RA). Figure S4: UPLC–MS chromatogram of a methanolic extract of *Z. noltei* leaves. Figure S5: ^1^H NMR spectrum (500 MHz, CD_3_OD) of apigenin 7-sulfate (APS) isolated from *Z. noltei*.
