# Supplementary material for: Rosmarinic Acid and Flavonoids of the Seagrass Zostera noltei: New Aspects on Their Quantification and Their Correlation with Sunlight Exposure
Source: Plants (Basel). 2023 Dec 6;12(24):4078. doi: 10.3390/plants12244078 (PMC10748107; doi:10.3390/plants12244078)
Supplement: Supplementary file 1 [file plants-12-04078-s001.zip › plants-2739921-supplementary.pdf]

Supplementary material

Rosmarinic Acid and Flavonoids of the Seagrass *Zostera noltei*: New Aspects on their Quantification and their Correlation with Sunlight Exposure

Isabel Casal-Porras [1], Kimberly Muñoz [1], María J. Ortega [2], Fernando G. Brun [1], and Eva Zubía [2,*]

[1] Departamento de Biología, Facultad de Ciencias del Mar y Ambientales, Universidad de Cádiz, 11510-Puerto Real (Cádiz), Spain; isabel.casal@uca.es (I.C.-P.), kimberly.munoz@uca.es (K.M.), fernando.brun@uca.es (F.G.B.)
[2] Departamento de Química Orgánica, Facultad de Ciencias del Mar y Ambientales, Universidad de Cádiz, 11510-Puerto Real (Cádiz) Spain; mariajesus.ortega@uca.es (M.J.O.)
* Correspondence: eva.zubia@uca.es (E.Z.)

Figure S1. Map of the Bay of Cadiz (Spain) and sites of *Z. noltei* sampling.
Table S1. Statistical data.
Table S2. Contents of phenolic metabolites (mg/g dw) in fresh, air-dried, freeze-dried, and frozen leaves of *Z. nolt*ei.
Figure S2. Scheme of the *in situ* experiment with *Z. noltei*.
Table S3. Contents of phenolic metabolites (mg/g dw) in leaves of *Z. nolt*ei under an UV-filter during 10, 30, and 60 days
Figure S3. UV-Vis spectra of a methanolic extract of *Z. noltei* leaves and of rosmarinic acid (RA).
Figure S4. UPLC-MS chromatogram of a methanolic extract of *Z. noltei* leaves.
Figure S5. ^{1}H-NMR spectrum (500 MHz, CD_3OD) of apigenin 7-sulfate (APS) isolated from *Z. noltei*.

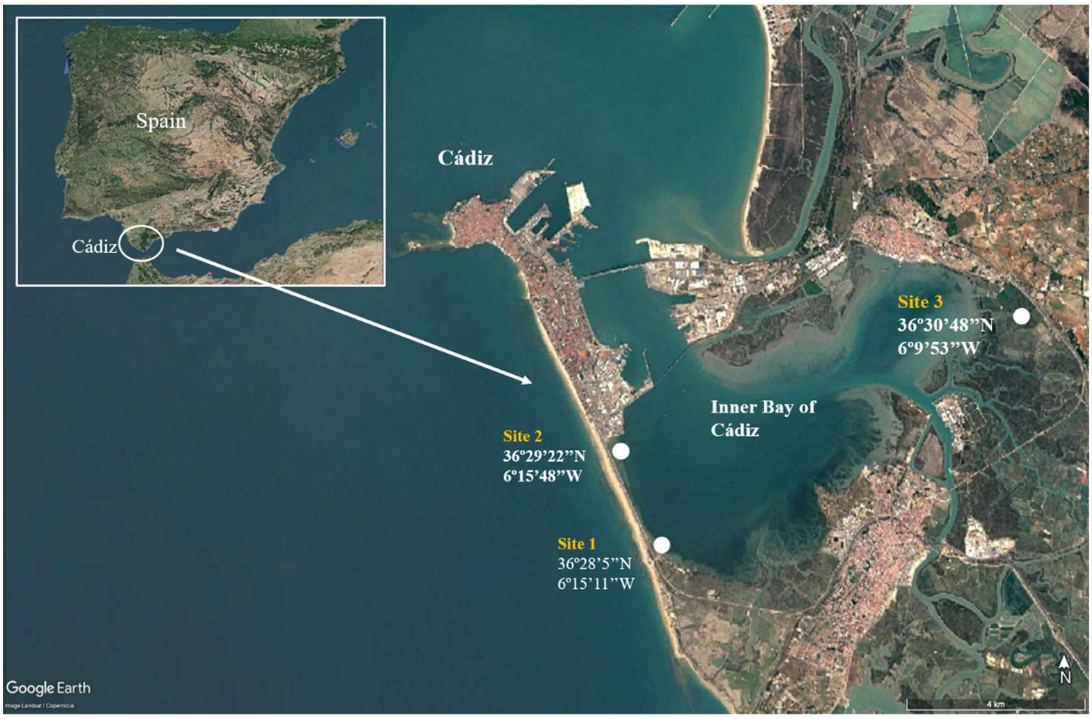

Figure S1. Map of the Bay of Cadiz (Spain) and sites of *Z. noltei* sampling (white circles).

Table S1. Results of the one-way ANOVA and Kruskal-Wallis test examining the differences between phenolic metabolites: rosmarinic acid (RA), apigenin 7-sulphate (APS), luteolin 7-sulphate (LS), diosmetin 7-sulphate (DS), apigenin 7-glucoside (APG), and luteolin 7-glucoside (LG). Bold numbers indicate significant differences at $p < 0.05$.

fresh *vs* air-dried *vs* freeze-dried *vs* frozen - January				
**One-way ANOVA test**	**df**	**MS**	***F***	***p***
Total flavonoid	3	556.7	145.4	**<0.05**
APG	3	0.7122	30.69	**<0.05**
LS	3	11.606	35.34	**<0.05**
APS	3	47.77	13.24	**<0.05**
**Kruskal-Wallis test**		**df**	**X^2**	***p***
RA		3	10.385	**<0.05**
LG		3	10.532	**<0.05**
DS		3	9.4615	**<0.05**
fresh *vs* air-dried *vs* freeze-dried *vs* frozen - March				
**One-way ANOVA test**	**df**	**MS**	***F***	***p***
Total flavonoid	3	206.40	29.58	**<0.05**
DS	3	84.04	28.22	**<0.05**
**Kruskal-Wallis test**		**df**	**X^2**	***p***
RA		3	9.2564	**<0.05**
LG		3	9.5595	**<0.05**
APG		3	10.458	**<0.05**
LS		3	9.0513	**<0.05**
APS		3	9.359	**<0.05**
zone-I *vs* zone-S - March				
**One-way ANOVA test**	**df**	**MS**	***F***	***p***
RA	1	880.5	75.91	**<0.05**
Total flavonoid	1	501.6	29.93	**<0.05**
APS	1	98.38	16.5	**<0.05**
DS	1	34.51	100.5	**<0.05**
APG	1	26.049	34.41	**<0.05**
LS	1	1.4145	2.335	0.157
zone-I *vs* zone-S - April				
**One-way ANOVA test**	**df**	**MS**	***F***	***p***
APG	1	4.477	11.35	**<0.05**
**Kruskal-Wallis test**		**df**	***X^2***	***p***
RA		1	8.3077	**<0.05**
Total flavonoid		1	7.8801	**<0.05**
APS		1	0.6410	0.423
DS		1	2.5641	0.109
LG		1	8.3368	**<0.05**
LS		1	2.1421	**<0.05**

control *vs* UV-Filter				
**One-way ANOVA test**	**df**	**MS**	***F***	***p***
RA, Day 10	1	35.15	2.144	0.193
RA, Day 30	1	246.75	10.18	**<0.05**
RA. Day 60	1	485.5	14.82	**<0.05**
**One-way ANOVA test**	**df**	**MS**	***F***	***p***
Total flavonoid, Day 10	1	4.867	0.637	0.455
**Kruskal-Wallis test**		**df**	X^2	***p***
Total flavonoid, Day 30		1	0.7590	0.383
Total flavonoid, Day 60		1	1.3333	0.248
**One-way ANOVA test**	**df**	**MS**	***F***	***p***
APG, Day 10	1	0.0200	0.567	0.480
APG, Day 30	1	0.2415	1.65	0.246
APG, Day 60	1	0.3486	1.894	0.218
**One-way ANOVA test**	**df**	**MS**	***F***	***p***
APS, Day 10	1	2.928	1.022	0.351
APS, Day 30	1	0.312	0.087	0.779
APS, Day 60	1	5.040	2.097	0.198
**One-way ANOVA test**	**df**	**MS**	***F***	***p***
DS, Day 10	1	0.3486	0.432	0.535
DS, Day 30	1	0.1105	0.476	0.516
DS, Day 60	1	0.2450	0.296	0.606
**Kruskal-Wallis test**		**df**	X^2	***p***
LG, Day 10		1	1.7943	0.180
LG, Day 30		1	0.3835	0.535
LG, Day 60		1	0.7875	0.374
**One-way ANOVA test**	**df**	**MS**	***F***	***p***
LS, Day 10	1	0.1081	0.045	0.839
LS, Day 30	1	0.288	0.32	0.592
LS, Day 60	1	0.042	0.023	0.885
Antioxidant activity				
**Kruskal-Wallis test**		**df**	X^2	***p***
Antioxidant activities (Zone-I *vs* Zone-S)		1	3.857	**<0.05**
**One-way ANOVA test**	**df**	**MS**	***F***	***p***
Antioxidant activities (Control *vs* UV-Filter)	1	357.0	8.878	**<0.05**

Table S2. Content (mg/g dw) of rosmarinic acid (RA), sulfated flavonoids APS, LS, DS, flavonoid glycosides LG, APG, and total flavonoids in fresh, air-dried, freeze-dried, and frozen leaves of *Z. noltei* collected in (**A**) January and (**B**) March. Values are mean ± SE (n= 3).For each compound different letters indicate significant differences (p<0.05, Table S1).

(A)

**Compound**	**fresh**	**air-dried**	**freeze-dried**	**frozen**
RA	29.99 ± 1.59^{a}	2.48 ± 0.24^{b}	17.67 ± 1.58^{c}	1.36 ± 0.17^{b}
APS	12.98 ± 1.46^{a}	4.28 ± 0.31^{b}	7.56 ± 1.37^{bc}	11.76 ± 0.83^{ac}
LS	5.51 ± 0.36^{a}	0.86 ± 0.05^{b}	2.35 ± 0.46^{b}	2.21 ± 0.31^{b}
DS	3.93 ± 0.20^{a}	0.96 ± 0.05^{b}	2.79 ± 0.61^{a}	4.04 ± 0.25^{a}
APG	1.26 ± 0.04^{a}	0.35 ± 0.02^{b}	0.65 ± 0.08^{b}	1.37 ± 0.15^{a}
LG	0.53 ± 0.03^{a}	nq	0.30 ± 0.02^{b}	0.42 ± 0.01^{c}
Total flavonoids	24.22 ± 1.96^{a}	6.45 ± 0.39^{b}	13.66 ± 2.52^{bc}	19.81 ± 1.39^{ac}

nq=not quantified, below limit of quantification.

(B)

**Compound**	**fresh**	**air-dried**	**freeze-dried**	**frozen**
RA	30.12 ± 0.40^{a}	4.56 ± 0.71^{b}	6.00± 1.01^{b}	31.98 ± 1.00^{a}
APS	11.74 ± 1.42^{a}	3.12 ± 0.19^{b}	4.24 ± 0.56^{b}	12.50 ± 1.48^{a}
LS	3.88 ± 0.62^{a}	0.65 ± 0.07^{b}	0.97 ± 0.13^{b}	3.52 ± 0.26^{a}
DS	2.92 ± 0.20^{a}	0.44 ± 0.14^{b}	1.06 ± 0.19^{b}	2.09 ± 0.32^{a}
APG	1.33 ± 0.20^{a}	0.16 ± 0.01^{b}	0.34 ± 0.04^{bc}	0.79 ± 0.02^{c}
LG	0.45 ± 0.03^{a}	nq	0.18 ± 0.01^{b}	0.43 ± 0.02^{a}
Total flavonoids	20.31 ± 2.11^{a}	4.36 ± 0.31^{b}	6.79 ± 0.87^{b}	19.34 ± 2.00^{a}

nq=not quantified, below limit of quantification.

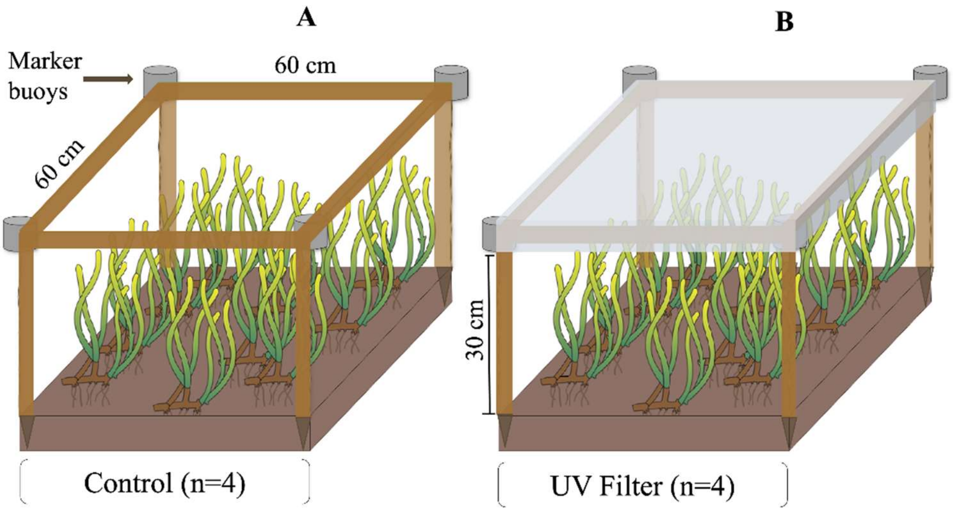

Figure S2. Scheme of the *in situ* experiment with *Z. noltei*: (**A**) control plants; (**B**) plants under an UV-filter.

Table S3. Content (mg/g dw) of rosmarinic acid (RA), sulfated flavonoids APS, LS, DS, flavonoid glycosides LG, APG, and total flavonoids in leaves of *Z. noltei* under a UV-filter for 10, 30, and 60 days and the corresponding controls. Values are mean ± SE (n= 4). For each day and compound different letters indicate significant differences (p<0.05, Table S1).

	**Day 0**	**Day 10**		**Day 30**		**Day 60**	
**Compound**		**control**	**UV-filter**	**control**	**UV-filter**	**control**	**UV-filter**
RA	30.35 ± 1.34	24.25 ± 2.31	20.06 ± 1.70	38.16 ± 3.37[a]	27.06 ± 0.88[b]	40.02 ± 2.05[a]	24.43 ± 3.49[b]
APS	12.58 ± 2.28	12.48 ± 0.23	13.69 ± 1.17	11.68 ± 1.31	11.29 ± 0.29	16.44 ± 0.96	14.86 ± 0.53
LS	3.25 ± 0.52	4.20 ± 0.89	4.43 ± 0.63	2.50 ± 0.41	2.88 ± 0.53	4.20 ± 0.80	4.06 ± 0.53
DS,	0.86 ± 0.16	1.45 ± 0.23	1.72 ± 0.44	0.63 ± 0.24	0.40 ± 0.25	1.17 ± 0.43	0.82 ± 0.48
APG	0.59 ± 0.06	0.81 ± 0.10	0.71 ± 0.09	0.82 ± 0.26	0.47 ± 0.08	1.15 ± 0.27	0.73 ± 0.14
LG	0.18 ± 0.10	0.25 ± 0.08	0.20 ± 0.07	0.14 ± 0.08	0.12 ± 0.07	0.26 ± 0.09	0.27 ± 0.02
Total Flav	17.47 ± 2.33	19.20 ± 1.45	20.76 ± 1.31	15.77 ± 1.90	15.16 ± 0.60	23.22 ± 1.17	20.73 ± 1.38

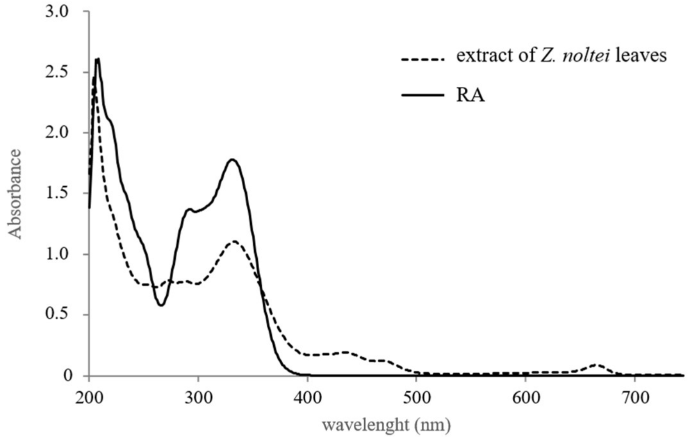

Figure S3. UV-Vis spectra of a methanolic extract of fresh leaves of *Z. noltei* and of rosmarinic acid (RA).

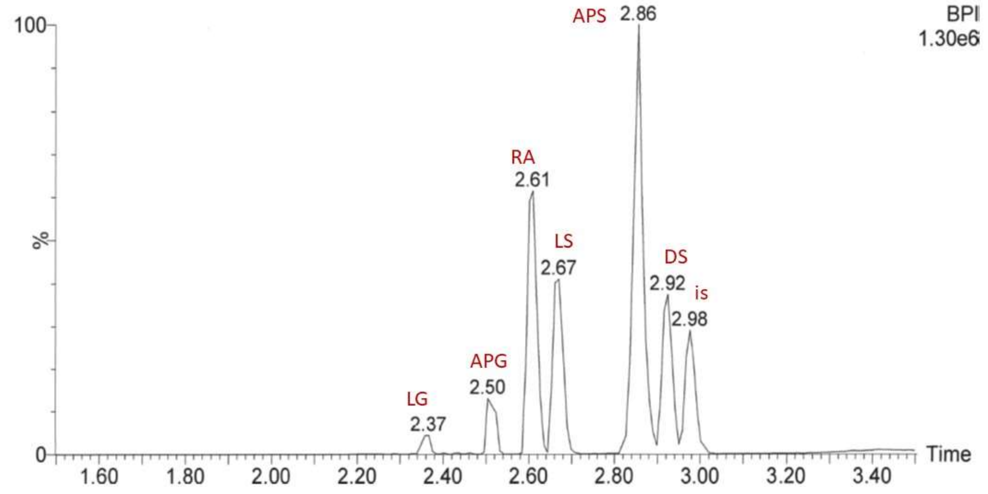

Figure S4. UPLC-MS chromatogram of a methanolic extract of fresh leaves of *Z. noltei*.
LG: luteolin 7-glucoside; APG: apigenin 7-glucoside; RA: rosmarinic acid; LS: luteolin 7-sulfate; APS: apigenin 7-sulfate; DS:diosmetin 7-sulfate; is:internal reference.

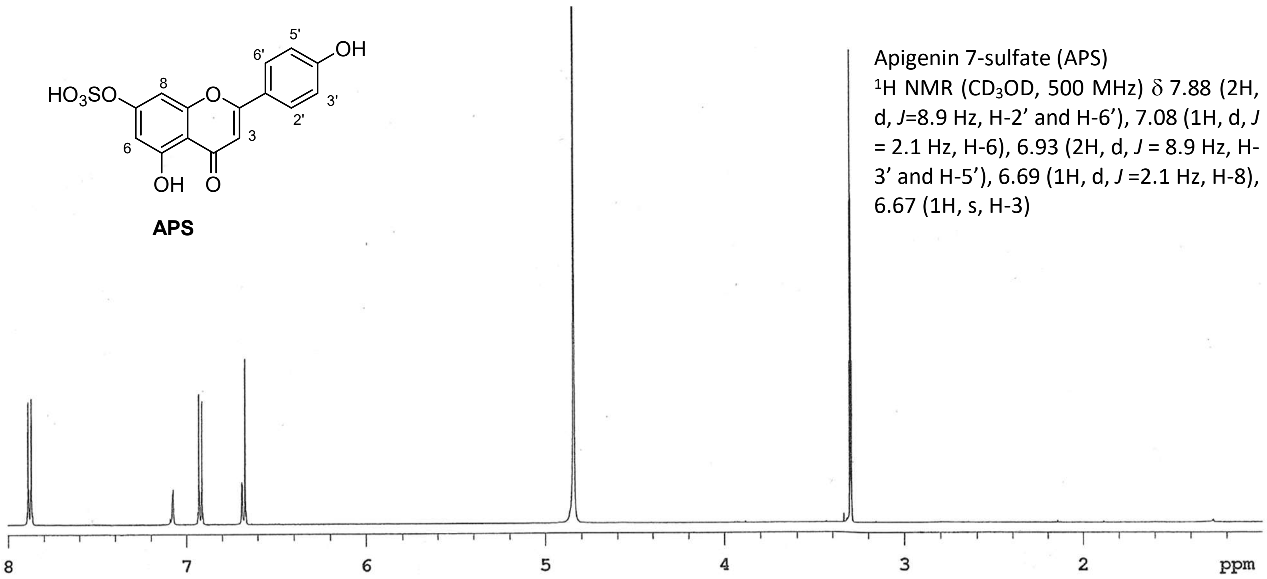

Figure S5. ^{1}H NMR spectrum (CD_3OD, 500 MHz) of apigenin 7-sulfate (APS) isolated from *Z. noltei* leaves.
